# Aggressive B-Cell Lymphomas

**DOI:** 10.1155/2012/604603

**Published:** 2012-03-14

**Authors:** Kikkeri N. Naresh, Ian Magrath, Martine Raphael, Lorenzo Leoncini

**Affiliations:** ^1^Department of Pathology, Imperial College Healthcare NHS Trust, London W12 OHS, UK; ^2^International Network for Cancer Treatment and Research, B1180 Brussels, Belgium; ^3^Service d'Hématologie et Immunologie Biologiques, Cytogénétique, Traitement de l'Hémophilie et Thérapie Transfusionnelle, 94275 Le Kremlin-Bicêtre Cedex, France; ^4^Department of Pathology, University of Siena, 53100 Siena, Italy

Aggressive B-cell lymphomas (ABLs) are a group of B-cell malignancies, that is, a relatively common form of cancer across the world. This issue on aggressive B-cell lymphomas compiles 12 exciting manuscripts, most of which are very meticulously performed reviews of the available current literature.

Three areas of lymphoma pathology and biology that are leading to a greater understanding of relatively recently defined entities are discussed. These include anaplastic lymphoma kinase (ALK)-positive large B-cell lymphoma (ALK+ LBCL), and the so-called grey zone lymphomas or lymphomas with intermediate features and the posttransplant lymphoproliferative disorders. ALK+ LBCL are characterised by gene rearrangement involving clathrin and *ALK* genes (t(2;17)(p23;q23)). Grey zone lymphomas include two unclassifiable B-cell lymphomas, one with features intermediate between diffuse large B-cell lymphoma (DLBCL) and classical Hodgkin lymphoma (cHL) and the second with features intermediate between DLBCL and Burkitt lymphoma (BL).

The biology of common lymphomas is addressed in two manuscripts. M. Rossi et al. review the current literature on molecular subsets of DLBCL, the distinction between DLBCL and BL, and gene expression characteristics of the grey zone lymphomas. This paper is accompanied by M. Cacciatore et al.'s manuscript that explores the importance of the microenvironment in lymphoma biology.

Two of the manuscripts deal with those aspects of endemic BL pathogenesis that have hitherto not been explored in great depth. The paper by S. Mannucci et al. explore the possibility of *Euphorbia tirucalli* acting as a cofactor in BL. They show that, in lymphoblastoid cell lines, *E. tirucalli* can modulate the EBV latency genes, result in polysomy for chromosome 8, and cause upregulation of c-MYC. Furthermore, Dr. C. van den Bosch raises question about a possible role for RNA viruses in the pathogenesis of BL.

Dr. I. Magrath reviews the evolution of BL treatment in Africa—from cyclophosphamide monotherapy to multiagent combination chemotherapy including central nervous system prophylaxis using combinations of cyclophosphamide, vincristine, prednisone, and methotrexate. He also highlights the benefits of collaboration between resource-rich and resource-poor settings. P. Vishnu and D. M. Aboulafia review how the treatment of HIV-associated lymphomas has evolved since the introduction of highly active antiretroviral therapy (HAART). The use of rituximab, in conjunction with multiagent chemotherapy, and where required high-dose chemotherapy (HDCT) and autologous stem cell rescue (ASCT) have all contributed to the success of treating HIV lymphomas. Treatment of ABL is further reviewed in two paper—one on ASCT in DLBCL and the other on novel therapies by K. Foon et al. To complete the issue, S. Donnou reviews the currently available murine models useful for studying lymphomagenesis, the lymphoma microenvironment, and the efficacy of new therapies.

By compiling these paper, we hope to enrich our readers and researchers with respect to these particularly common, yet usually highly treatable aggressive B-cell malignancies.

*Kikkeri N. Naresh*

*Ian Magrath*

*Martine Raphael*

*Lorenzo Leoncini*

